# Characterization of a novel gene, Lsa(F), conferring resistance to pleuromutilins, lincosamides and streptogramin A in *Streptococcus* *parasuis*

**DOI:** 10.1186/s13567-026-01784-0

**Published:** 2026-07-07

**Authors:** Xingyang Dai, Paulin Mungongo Mayama, Xiao Liu, Xiao Gao, Jingyan Xu, Xiaoming Wang, Jinhu Huang, Zongfu Wu, Diafuka Saila-Ngita, Liping Wang

**Affiliations:** 1 MOE Joint International Research Laboratory of Animal Health and Food Safety, College of Veterinary Medicine, Nanjing Agricultural University, Nanjing, China; 2 Sanya Institute of Nanjing Agricultural University, Sanya, China; 3 Risk Assessment Center of Veterinary Drug Residue and Antimicrobial Resistance, Nanjing Agricultural University, Nanjing, China; 4 Faculty of Veterinary Medicine, University of Kinshasa, Kinshasa, Democratic Republic of Congo

**Keywords:** Lsa(F), PLS_A_ resistance, *Streptococcusparasuis*, mobile genetic elements

## Abstract

Transferable antimicrobial resistance poses significant threats to public health. This issue is further exacerbated by Lsa homolog-mediated resistance to pleuromutilins, lincosamides, and streptogramin A (PLS_A_). This study aimed to characterize a novel PLS_A_ resistance gene, designated Lsa(F), identified in *Streptococcus* *parasuis*. The Lsa(F) protein exhibits 41.9–58.7% sequence identity with known Lsa variants and shares structural features typical of the F lineage of the ATP-binding cassette (ABC) protein superfamily that confers ribosome protection. Functional expression of Lsa(F) in *Streptococcus* *suis* and *Staphylococcus* *aureus* confers resistance to PLS_A_. Although the prevalence of the Lsa(F) gene among *S*. *parasuis* isolates was found to be relatively low, database analyses revealed that this gene is primarily present in *Streptococcus* and *Lactococcus* species. In *Streptococcus*, the Lsa(F) gene is predominantly associated with various mobile genetic elements (MGEs), including plasmids, integrative and conjugative elements (ICE), and ICE-derived elements; in *Lactococcus*, it is primarily located on plasmids and at a specific chromosomal locus. Examination of the genetic context analysis identified the insertion sequence IS*Lll1* downstream of Lsa(F) on almost all chromosomal MGEs and plasmids, suggesting a potential mechanism facilitating its movement. Notably, our results indicate that the Lsa(F) gene can be transferred from *S. parasuis* to *Streptococcus* *suis*, highlighting a risk of its spread to other pathogens. Therefore, it is necessary to enhance surveillance and epidemiological monitoring of the Lsa(F) gene to reduce the potential public health risks associated with its spread.

**Supplementary Information:**

The online version contains supplementary material available at 10.1186/s13567-026-01784-0.

## Introduction

Antimicrobial resistance (AMR) poses a grave threat to global public health, undermining the efficacy of conventional therapies and leading to increased treatment failures and mortality [1, 2]. Among the most alarming are the pathogens designated by the World Health Organization (WHO, 2024) as priority pathogens [3], such as carbapenem-resistant *Enterobacterales* (CRE), vancomycin-resistant enterococci (VRE), and methicillin-resistant *Staphylococcus aureus* (MRSA). To combat these formidable pathogens, last-resort antimicrobial agents including colistin against CRE and linezolid (oxazolidinones) against Gram-positive bacteria, serve as critical therapeutic options [4, 5]. While tiamulin and valnemulin are pleuromutilin derivatives extensively employed in veterinary medicine, particularly for swine and poultry [6], subsequent structural modifications of the pleuromutilin have yielded novel agents such as retapamulin and lefamulin, which are now approved for clinical use in human against Gram-positive bacterial infections [7, 8]. This trajectory from veterinary to human applications underscores the critical and expanding role of pleuromutilins in anti-infective therapy. However, the dual application of pleuromutilins in human and animal health may facilitate the selection and dissemination of resistance genes, presenting a substantial public health risk.

A key mechanism of resistance to these critically important drugs, including pleuromutilins, oxazolidinones, and streptogramins, is mediated by ATP-binding cassette (ABC)-F family proteins (ABC-F) [9, 10]. These proteins confer resistance through a ribosome protection, utilizing ATP hydrolysis to displace the bound antibiotic from the ribosome without modifying the drug or the target site[11]. Several members of the ABC-F family protein have been identified, such as Msr, Vga, and Lsa, along with OptrA and PoxtA [12, 13]. Notably, the genes encoding these proteins are often located on mobile genetic elements (MGEs) [14, 15], including plasmids, prophages, integrative and conjugative elements (ICEs), and transposons, thereby facilitating the horizontal transfer of resistance determinants among pathogenic bacteria. Structurally, despite considerable sequence diversity, all ABC-F proteins share a conserved architecture featuring two nucleotide binding domains (NBDs) connected by a linker region of approximately 80 amino acids with variable composition, and they lack transmembrane domains (TMDs) [10]. Furthermore, the increasing identification of novel ABC-F proteins across diverse bacterial species in recent years [16–18] reflects their ongoing evolutionary diversification and suggests the widespread distribution of distinct variants within bacterial populations.

In this study, we identified a multidrug-resistant *Streptococcus* *parasuis* strain exhibiting resistance to tiamulin in the absence of any known resistance mechanism, suggesting the involvement of a novel resistance determinant. Subsequent investigations revealed that tiamulin resistance is conferred by an ABC-F family gene (previously noted as Lsa_var2), which had been briefly mentioned in a prior bioinformatic analysis but lacked experimental validation [19]. We confirmed that this gene confers a combined resistance phenotype to pleuromutilins, lincosamides, and streptogramin A (PLS_A_), and have formally designated it Lsa(F). This finding not only expands the known repertoire of ABC-F-mediated resistance mechanisms, but also highlights the potential emergence and spread of such genes, particularly under the cross-selection pressure exerted by the shared use of pleuromutilins in human and veterinary medicine.

## Materials and methods

### Bacterial strains

A total of 26 clinical isolates of *S. parasuis* were used in this study (Additional file 1) and were identified by polymerase chain reaction (PCR) using primers as previously described [20, 21] (Additional file 2). Among them, the strain SFJ45, isolated in 2017 from a swine nasal swab, has been previously characterized with respect to its antimicrobial susceptibility profile and resistome on the basis of the presence of known resistance genes [22]. *Escherichia coli* DH5α, *Streptococcus suis* P1/7, and *Staphylococcus* *aureus* RN4220 were used as hosts for cloning of the Lsa(F) gene (Additional file 2).

### Antimicrobial susceptibility testing

The minimal inhibitory concentrations (MICs) of tiamulin, valnemulin, lefamulin, retapamulin, lincomycin, clindamycin, virginiamycin M1, and florfenicol were determined by the broth microdilution method using custom-prepared susceptibility plates (tested range 0.25–128 mg/L). The results were interpreted according to the Clinical and Laboratory Standard Institute (CLSI) document M100 ED36th [23]. *S. aureus* ATCC 29213 was used as the quality control strain.

### Prevalence of Lsa(F) in clinical strains

To evaluate the prevalence of the Lsa(F) gene among clinical isolates, PCR screening was performed on 26 *S. parasuis* isolates obtained from our laboratory collection. Furthermore, the presence of resistance genes Lsa(E), *cfr*, and *cfr*(D), which are commonly found in *S. parasuis* and linked to decreased susceptibility to tiamulin [24], was also investigated. The primer sequences [25–27] and PCR conditions for detection of the Lsa(E), *cfr*, and *cfr*(D) genes are shown in Additional file 2.

### Bacteria construction

A recombinant vector for the Lsa(F) gene was constructed using the shuttle vector pSET2 [28]. Briefly, a fragment containing the complete open reading frame of the Lsa(F) gene plus a ~500 bp that contains the Lsa(F) own promoter was amplified from the strain SFJ45 genomic DNA. The purified fragment was then ligated into the pre-linearized vector pSET2 through the *In-Fusion* cloning technique, resulting in the recombinant vector pSET2-Lsa(F). The vector was then transformed into *E. coli* DH5α for propagation and verification, followed by electro-transformation into competent cells of *S. suis* P1/7 and *S. aureus* RN4220. Transformants were selected on tryptic soy agar (TSA) plates containing 100 mg/L spectinomycin and were verified through PCR amplification of the Lsa(F) gene. Primers and PCR conditions are shown in Additional file 2.

### Bioinformatics analysis and genetic context of Lsa(F)

The complete genome sequence of *S. parasuis* SFJ45, the focal strain in this study, was previously determined [22]. To investigate the phylogenetic relationship of the ABC-F protein encoded by Lsa(F), a maximum‑likelihood phylogenetic tree was constructed using MEGA X, incorporating Lsa (F) and other ABC‑F family proteins associated with antibiotic resistance. Amino acid sequence identities between Lsa(F) and known Lsa variants were determined via BLASTP alignment.

To assess the prevalence of Lsa(F) among streptococci, we initially screened the genome sequences of strains available in our laboratory collection. In addition, Lsa(F)-positive strains available in the GenBank database (last accessed 15 March 2025) were incorporated into the analysis, focusing on highly similar complete nucleotide sequence hits with Lsa(F) in strain SFJ45 that exhibited both coverage and identity thresholds exceeding 80%.

To characterize the genetic context of Lsa(F), 50 kb regions flanking the identified Lsa(F) locus were examined, a range deemed adequate for predicting the majority of MGEs [29]. Detection of ICEs and other elements within these regions was performed using ICEberg 3.0 [30]. Insertion sequence (IS) elements were identified by ISfinder [31], while acquired antimicrobial resistance genes (ARGs) were identified through ResFinder (version 4.7.2) with a threshold of > 80% identity and a minimum length of 60 bp [32]. Comparative analysis and visualization of the genetic contexts encompassing Lsa(F) were carried out using EasyFig 2.2.2 [33].

Given the frequent misidentification of *S*. *parasuis* in earlier studies, we reevaluated the taxonomic classification of Lsa(F)-positive strains designated as *S*. *suis*. A core‑genome‑based phylogenetic tree was constructed using the Bacteria Genome Tree tool available on the Bacterial and Viral Bioinformatics Resource Center (BV-BRC) website [34].

### Transfer experiments

Conjugative transfer was conducted using a filter mating assay as described previously [35]. The sequenced Lsa(F)-positive *S. parasuis* clinical strains served as the donors, while *S. suis* strain P1/7RF (rifampicin and fusidic acid resistance) was employed as the recipient. Transconjugants were selected on TSA plates supplemented with 50 mg/L rifampicin, 50 mg/L fusidic acid, and 8 mg/L tiamulin (or 4 mg/L lincomycin), and their identity was subsequently confirmed via PCR analysis (Additional file 2). Transfer frequency was determined as the average number of transconjugants per donor cell across three independent replicates.

## Results

### Characterization of the Lsa(F) gene

**Figure 1:**
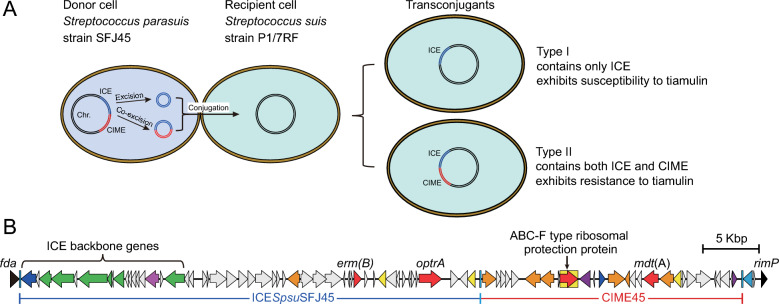
**The discovery of the Lsa(F) gene.** **A** Schematic presentation of the conjugal transfer mechanism of tiamulin resistance determinants. **B** Schematic presentation of tandem elements ICE*Spsu*SFJ45 and CIME45 in *Streptococcus* *parasuis* strain SFJ45 (CP102747) and the location of the Lsa(F) gene (ABC-F type ribosomal protection protein). The positions and transcriptional directions of the open reading frames (ORFs) are denoted with arrows.

*S. parasuis* strain SFJ45 exhibits resistance to tiamulin; however, it lacks known resistance determinants or point mutations in the *23S rRNA*, *rplC*, and *rplD* genes [7]. In transfer experiments, the use of lincomycin as a resistance selection marker in a filter membrane conjugation assay revealed that the tiamulin resistance was transferred to a subset of recipient cells (Figure 1A, Additional file 3). Whole-genome analysis showed that strain SFJ45 contains tandem elements at the *fda* locus, namely ICE*Spsu*SFJ45 and a *cis*-mobilizable element (CIME), CIME45 (Figure 1B). ICE*Spsu*SFJ45 harbors the resistance genes *optrA* and *erm*(B), while CIME45 carries the resistance gene *mdt*(A). Notably, resistance to tiamulin was observed only the transconjugants for which a co-transferred of the ICE and the CIME, whereas the ICE alone did not demonstrate such resistance. This finding suggests the presence of an unknown resistance determinant within the CIME45 element.

**Figure 2:**
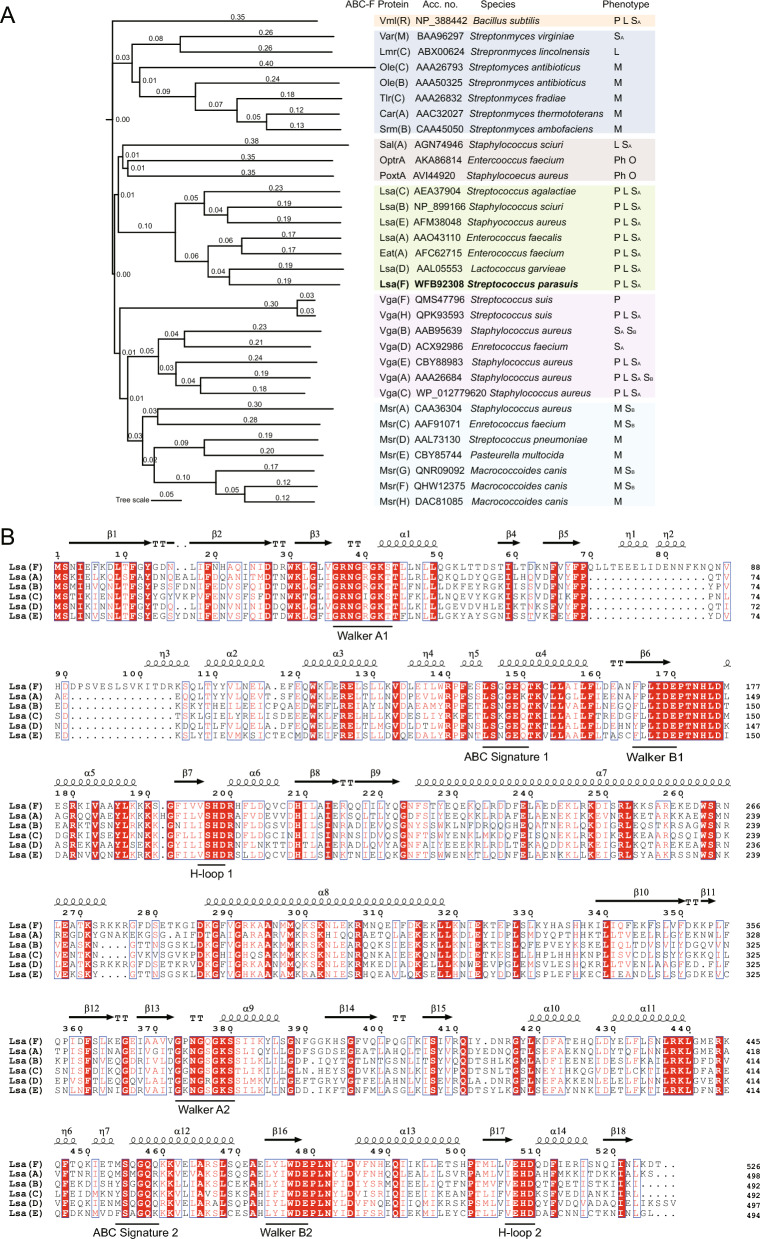
**Identification of the resistance gene Lsa(F)**. **A** Phylogenetic tree of ABC-F proteins constructed using the maximum likelihood method. The GenBank accession numbers of the ABC-F proteins are indicated. **B** Multiple alignment of Lsa variant proteins. Two copies of the Walker A and Walker B motifs as well as ABC signatures and H-loops are shown in underline.

An analysis of the CIME45 element identified a gene annotated as encoding an ABC-F type ribosomal protection protein comprising 526 amino acids. This gene corresponds to a Lsa variant (Lsa_var2), which has been briefly mentioned in previous study but has not been experimental validation [19]. Therefore, it is postulated that this gene confers transferable resistance to tiamulin in host cells. A neighbor-joining phylogenetic tree was constructed to evaluate the relationships of this protein with other antibiotic-resistance ABC-F family proteins (Figure 2A), indicating its association with a lineage that includes Lsa homologues and Eat(A). Sequence alignment revealed that the protein shares 41.9–58.7% amino acid identity with previously characterized Lsa variant proteins (Additional file 4). Structurally, the protein exhibits the canonical features typical of ABC-F family members, including two Walker A motifs, two Walker B motifs, two ABC signature motifs, and two H-loop switches, while lacking a transmembrane-associated domain (Figure 2B). Its catalytic motif, LSGGQ, is conserved and consistent with that found in the majority of ABC superfamily proteins [12]. On the basis of these characteristics, this gene has been designated as a novel gene, termed Lsa(F), in accordance with the nomenclature system for macrolide, lincosamide, and streptogramin (MLS) resistance genes.

### Resistance phenotype conferred by the Lsa(F) gene in *S*. *suis* and *S*. *aureus*

We hypothesized that the Lsa(F) gene may be responsible for conferring resistance to tiamulin. To investigate this, the gene was cloned into the pSET2 vector and transformed into two bacterial species, and then we observed tiamulin resistance in *S. suis* and *S. aureus* (Table 1). Minimum inhibitory concentration (MIC) assays revealed that acquisition of the Lsa(F) gene resulted in at least an eightfold increase in MIC values against pleuromutilin antibiotics in the transformants. Notably, *S. suis* P1/7 and *S. aureus* RN4220 exhibited a 128-fold increase in MIC for tiamulin. Furthermore, retapamulin and lefamulin, which are only approved for clinical application in human medicine, displayed a 64-fold MIC increase in both *S. suis* and *S. aureus* strains. The transformants also showed elevated MICs for lincomycin, clindamycin, and virginiamycin M_1_, with minimum increases of at least 32-fold and 4-fold, respectively. These findings suggest that the novel Lsa(F) gene confers a PLS_A_ resistance phenotype in bacterial cells, consistent with phenotypes associated with other Lsa gene variants [36, 37].

**Table 1:** **Antimicrobial susceptibilities of strains carrying a cloned copy of the** **Lsa(F) gene**

Strain	Minimal inhibitory concentration (mg/L)							
	LEF	RET	TIA	VAL	LIN	CLI	VGM	FFC
*S. parasuis* SFJ45 (WT)	64	64	128	128	> 128	> 128	16	64
*S. suis* P1/7	≤ 0.25	≤ 0.25	0.5	≤ 0.25	≤ 0.25	≤ 0.25	1	0.5
*S. suis* P1/7::pSET2	≤ 0.25	≤ 0.25	0.5	≤ 0.25	≤ 0.25	≤ 0.25	1	0.5
*S. suis* P1/7::pSET2-Lsa(F)	16	32	64	32	32	32	4	0.5
*S. aureus* RN4220	≤ 0.25	≤ 0.25	0.25	≤ 0.25	0.5	≤ 0.25	0.5	4
*S. aureus* RN4220::pSET2	≤ 0.25	≤ 0.25	0.25	≤ 0.25	0.5	≤ 0.25	0.5	4
*S. aureus* RN4220::pSET2-Lsa(F)	16	32	32	16	16	16	4	4

*LEF* lefamulin, *RET* retapamulin, *TIA* tiamulin, *VAL* valnemulin, *LIN* lincomycin, *CLI* clindamycin, *VGM* virginiamycin M_1_, *FFC* florfenicol, *WT* wild type

### Prevalence of the Lsa(F) gene

Among the 26 clinical isolates of *S. parasuis*, 19 strains had high MIC values (>16 mg/L) of tiamulin. PCR analysis showed the presence of the Lsa(F) gene in ten strains and the Lsa(E) gene in nine strains. The observed phenotypic resistance patterns were consistent with the genotypic data. Notably, the *cfr* gene is also present in two Lsa(F)-positive strains. The Lsa(E) gene encodes a homologous variant of the Lsa proteins, which mediates cross-resistance to PLS_A_ antibiotics, whereas the *cfr* gene encodes a ribosomal RNA methyltransferase that imparts resistance to phenicols, lincosamides, oxazolidinones, pleuromutilins, and streptogramin A (PhLOPS_A_) antibiotics. The Lsa(F)-positive strains were isolated from three provinces (Jiangsu, Zhejiang, and Guizhou) in China during the period spanning 2016–2025.

To expand the sample size, additional strains from public databases were included in the analysis (Additional file 5). The results showed that the Lsa(F) gene was present in a limited number of *Streptococcus* strains in the database, including five strains of *S*. *parasuis*, eight strains of *S*. *suis*, and two strains of *Streptococcus pluranimalium*. These strains were sourced from multiple geographical locations, including various provinces in China, as well as from Vietnam and Myanmar. However, genomic evolutionary analysis revealed that the Lsa(F)-positive *S. suis* strains were, in fact, *S*. parasuis (Additional file 6). The Lsa(F) gene was also detected in *Lactococcus* species, including 11 strains of *Lactococcus* *lactis*, 2 strains of *Lactococcus* *lactis* subsp. *lactis*, and 2 strains of *Lactococcus* sp. Overall, the Lsa(F) gene was found in both *Streptococcus* and *Lactococcus* species, but with a low prevalence, suggesting that it may be in the early stages of becoming more widespread.

Additionally, a gene similar to the Lsa(F) gene was identified (Additional file 7). The protein encoded by this Lsa(F)-like gene comprises 522 amino acids and shares 81.2% sequence identity with Lsa(F). Notably, this protein retains the same functional motif as Lsa(F), with the exception of the antibiotic resistance determinant loop. Further research is required to determine its potential role in conferring PLS_A_ resistance. This gene was found to be widely prevalent in *Lactococcus* *lactis* and *Lactococcus* *lactis* subsp. *lactis*, whereas its presence in *Lactococcus* *cremoris* was comparatively infrequent, being detected in only seven strains.

### Characterization of the Lsa(F) genetic context and its associated mobile genetic elements

**Figure 3:**
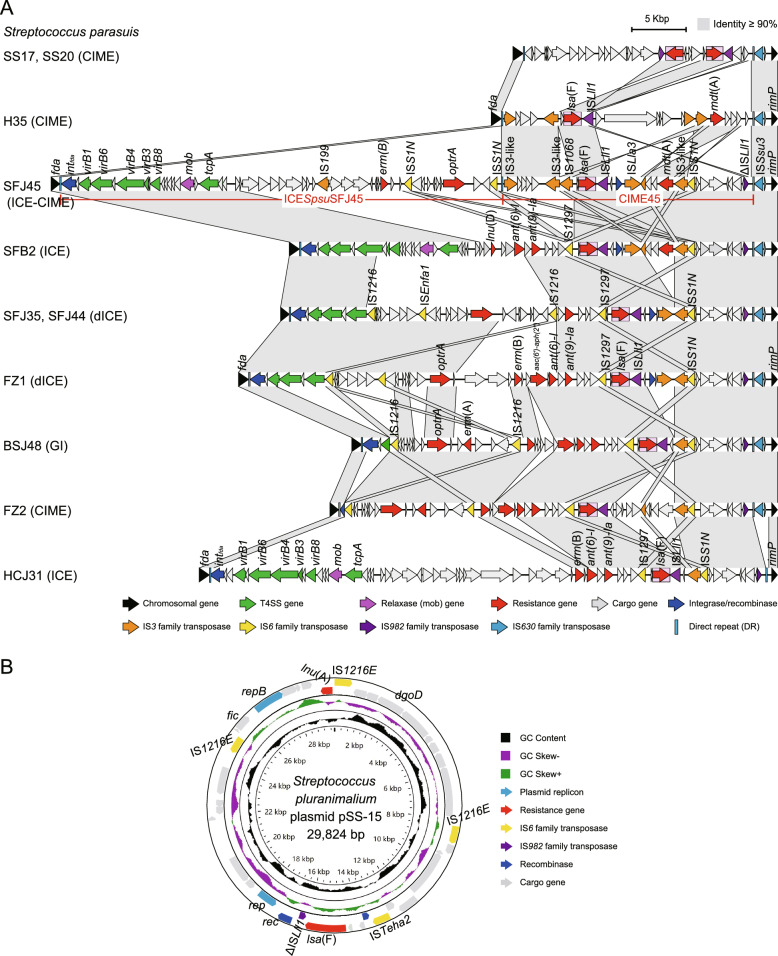
**The Lsa(F) gene in *Streptococcus* species**. This gene is located on diverse mobile genetic elements, either **A** integrated into the chromosomal background or **B** carried on plasmids. The ORFs are shown as arrows indicating the transcription direction. Homologous gene clusters in different strains are shaded in grey.

We conducted a thorough analysis of the genetic context surrounding the Lsa(F) gene. In *S. parasuis*, the majority of Lsa(F)-positive strains are associated with MGEs integrated within chromosomal backgrounds, including ICEs, CIMEs, and genomic islands (GIs), as well as defective ICEs (dICEs) characterized by partial deletions in their conjugation modules or the absence of relaxases [38] (Figure 3A). These genetic elements are primarily integrated at the *fda* locus, which is known as a hotspot for ICE*St3* family ICEs [39]. The Lsa(F)-carrying ICEs have a synthetic “core” structure that includes modules for recombination, conjugation, and regulation, which is similar to that of ICE*St3*, although with a lower nucleotide identity [22]. The variable regions of these elements exhibit similar genetic environments and are found in link with many ARGs and various IS elements, suggesting a potential role in antibiotic co-selection and IS-mediated movement [40]. Additionally, certain Lsa(F) sequences were identified on short contigs, ranging from 3103 bp to 146 972 bp, which did not provide enough information about their genetic backgrounds (Additional file 8). Notably, in strains 15368–09512 and 15339–02824, the Lsa(F) gene was located in fragments positioned upstream of the *SUT286_17930* locus and downstream of the *nemA* locus, respectively, suggesting the presence of a novel attachment site distinct from the *fda* locus.

In *S. pluranimalium*, the Lsa(F) gene in one strain, was found on a plasmid belonging to the RepB family, coexisting with *lnu*(A) (Figure 3B). In another strain, the Lsa(F) gene was located on a 7612 bp contig, with a Rep3 family plasmid replication initiation protein present upstream, suggesting that it may be a plasmid fragment (Additional file 8). Notably, *S*. *pluranimalium*, formerly identified as *S*. *suis* serotype 33 [41] and* S*. *parasuis*, previously classified as *S. suis* serotypes 20, 22, and 26 [42], are closely related to *S*. *suis* and represent potential opportunistic zoonotic pathogens [24, 43]. It is imperative to monitor the dissemination of the Lsa(F) gene within these species.

**Figure 4:**
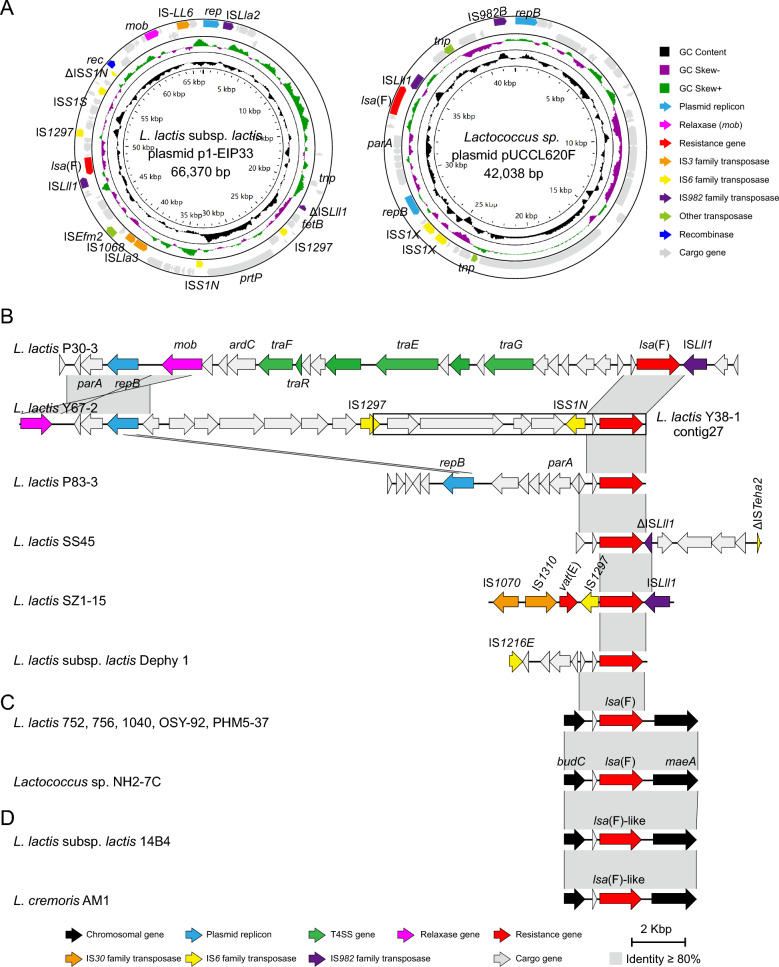
**The Lsa(F) gene in *Lactococcus* species**. The genetic context of this gene exhibits considerable variability, being capable of **A** being situated on plasmids, or **B** occurring within a contig that either contains or lacks a plasmid replicon, or **C** being integrated into the chromosome. **D** The Lsa(F)-like gene is located on the chromosome. The ORFs are shown as arrows indicating the transcription direction. Homologous gene clusters in different strains are shaded in grey.

In *Lactococcus* species, the Lsa(F) gene was identified on plasmids belonging to the RepX and RepB families in two strains, respectively, with no additional resistance genes (Figure 4A). The remaining Lsa(F) sequences were located on short contigs ranging from 5059 bp to 25 035 bp (Figure 4B). In three of these strains, the Lsa(F) gene was found on contigs measuring between 9546 bp and 25 035 bp, all positioned upstream of plasmid replication initiation proteins of RepB family, suggesting they represent plasmid fragments. Additionally, in certain strains, the Lsa(F) gene was found to be located between the *budC* and *maeA* genes and become immobilized on the chromosome following the loss of adjacent IS elements; the same pattern was observed for the Lsa(F)-like gene (Figure 4C, D).

**Figure 5:**
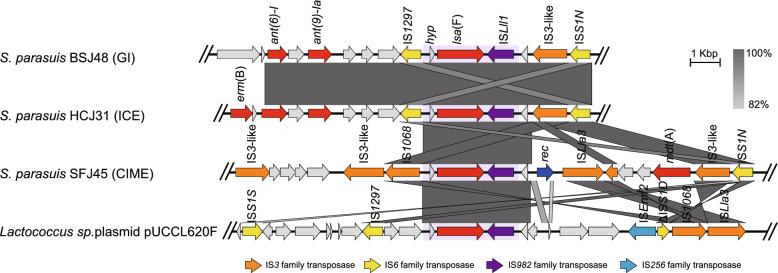
**The insertion sequence IS*Lll1* is consistently located downstream of the Lsa(F) gene**. The conserved genetic structure associated with the Lsa(F) gene consists of an upstream hypothetical protein gene (*hyp*), followed by the Lsa(F) gene, and subsequently the IS*Lll1* element, forming the structural unit *hyp*-Lsa(F)-IS*Lll1*. This organizational arrangement is consistently observed in all cases where the Lsa(F) gene is located on chromosomal MGEs or plasmids.

In some strains of *S. parasuis*, transposases belonging to the IS3 and IS6 families are frequently detected in the vicinity of the Lsa(F) gene, suggesting their potential role in the formation of composite transposons that may facilitate the movement of Lsa(F) (Figure 3A). Notably, in all strains where Lsa(F) is located on either chromosomal MGEs or plasmids, the IS element IS*Lll1* is consistently present downstream of the gene (Figure 5), except in case of short contigs resulting from limited sequencing coverage. This arrangement, together with an upstream gene encoding a hypothetical protein (*hyp*), constitutes a conserved 3135‑bp structural unit, arranged as *hyp–*Lsa (F)–IS*Lll1*. The IS*Lll1* element was 999 bp in length and contained an 891 bp DDE-type transposase of 296 amino acids. One base substitution was detected between the imperfect 20 bp downstream and upstream terminal inverted repeat (TIR) (upstream 5′- ACCCGAATTGCTAGTTAATT -3′ and downstream 5′- ACCCGAATTGCTAGTTGATT -3′), which is consistent with previously identified IS*Lll1* members. However, a typical target site duplication associated with IS*Lll1* had not been observed. IS*Lll1* is classified within the IS*982* family, which is commonly found in lactic acid bacteria and is known to play a role in the acquisition and dissemination of antibiotic resistance genes [44]. The members of the IS*982* family are capable of inserting into promoter regions or the coding sequence of antibiotic resistance or virulence genes, resulting in complete or partial activation/increase in expression or inactivation of the corresponding genes [44]. Notably, IS*Lll1* does not disrupt the integrity of the Lsa(F) open reading frame; instead, it is situated downstream of Lsa(F), suggesting that it may facilitate the transposition of Lsa(F).

### Transferability of Lsa(F) from *S. parasuis* to *S. suis*

Conjugation was successfully performed using three Lsa(F)-positive *S. parasuis* strains (HCJ31, SFB2, SFJ45), with *S. suis* strain P1/7RF serving as the recipient. PCR analysis confirmed the integration of the Lsa(F)-carrying elements into the *fda* locus of the recipient strain in all instances. All three transconjugants significantly decreased susceptibility to pleuromutilins, lincosamides, and streptogramin A (Table 2). In the donor strains, the Lsa(F) gene was located on an ICE in HCJ31 and SFB2, whereas in SFJ45, it was situated on a CIME, with transfer occurring via *cis* mobilization mediated by ICE at the same locus. In strains where conjugative transfer was unsuccessful, the Lsa(F) gene was found within dICE or ICE-derived GIs. Although host strains harbored ICE at other attachment sites, the absence of the origin of transfer site (*oriT*) limited transfer to the recipient.

**Table 2:** **Antimicrobial susceptibilities of** **Lsa(F)-positive** ***S. parasuis***, ***S. suis* P1/7RF, and the transconjugants**

Strain	Transfer frequency	Minimal inhibitory concentration (mg/L)							
		LEF	RET	TIA	VAL	LIN	CLI	VGM	FFC
Recipients									
*S. suis* P1/7RF		≤ 0.25	≤ 0.25	0.5	≤ 0.25	≤ 0.25	≤ 0.25	1	0.5
Donors									
HCJ31		32	64	64	64	> 128	> 128	8	16
SFB2		32	32	64	32	> 128	> 128	8	64
SFJ45		64	64	128	128	> 128	> 128	16	64
Transconjugants									
TC-HCJ31	(5.36 ± 3.18) × 10^−7^	16	32	16	32	> 128	> 128	8	0.5
TC-SFB2	(2.18 ± 4.23) × 10^−7^	16	32	32	16	128	128	4	0.5
TC-SFJ45-IC	(4.72 ± 2.71) × 10^−8^	32	32	64	32	64	32	8	32

Transfer frequency was calculated by CFUs of transconjugants/donors. *LEF* lefamulin, *RET* retapamulin, *TIA* tiamulin, *VAL* valnemulin, *LIN* lincomycin, *CLI* clindamycin, *VGM* virginiamycin M_1_, *FFC* florfenicol, *WT* wild type

### Discussion

The ABC-F family proteins are generally conferring resistance to one or more classes of antibiotics. For example, the OptrA and PoxtA proteins confer cross-resistance between florfenicol, an antibiotic exclusively utilized in animals, and linezolid, a critically important antibiotic for treating severe human infections [17, 18, 45]. The spread of these resistance determinants would pose a significant threat to public health security and animal husbandry. In the present study, we identified a novel member of the ABC-F family protein, termed Lsa(F). This protein represents a new variant within the Lsa homologs and confers resistance to three classes of anti-ribosomal antibiotics, including pleuromutilins, lincosamides, and streptogramin A.

With the acquisition of the Lsa(F) gene, host bacteria exhibited significantly reduced susceptibility to all four pleuromutilin antibiotics. Among these, retapamulin was approved for topical application in the USA in 2007, while lefamulin, recognized for its potent activity against multidrug-resistant *Streptococcus* *pneumoniae* and *S. aureus*, was approved for human systemic use in 2019 [7, 8]. In contrast, tiamulin and valnemulin are antibiotics exclusively utilized in veterinary medicine. [6]. Notably, the recently reported SrpA, now renamed Vga(H), in *S. suis* also confers resistance to PLS_A_ antibiotics and has been confirmed to confer resistance to all four aforementioned antibiotics in host bacteria [16]. The widespread clinical application of tiamulin and valnemulin in veterinary contexts imposes continuous selective pressure on the Lsa(F) gene, thereby promoting its persistence and dissemination among bacteria originating from animal sources. The results of the present study corroborate this observation, as strains harboring Lsa(F) were predominantly isolated from animal-derived samples. Nevertheless, the potential risk of horizontal transfer of Lsa(F) to human pathogens warrants careful consideration, given that such an event could undermine the clinical effectiveness of retapamulin and lefamulin in treating human infections.

Among the currently characterized variants of the Lsa gene, Lsa(E) exhibits the broadest distribution, having been identified in genera such as *Enterococcus* [46, 47], *Staphylococcus* [48, 49], *Streptococcus* [50, 51], and *Erysipelothrix rhusiopathiae* [52]. This gene is frequently associated with various MGEs, including plasmids, ICEs, prophages, GIs, and transposons. The present study revealed that the Lsa(F) gene is currently found predominantly within *Streptococcus* and *Lactococcus* species, with its distribution remaining relatively limited and possibly representing an early phase of dissemination. Opportunistic zoonotic pathogens such as *S. parasuis* and *S. pluranimalium* harbor Lsa(F) in close association with MGEs. This gene can be located on plasmids or integrated into chromosomal regions comprising ICEs, dICEs, and ICE derivatives including CIMEs and GIs. The close association of ARGs with various MGEs appears to be a prevalent phenomenon in *S. parasuis*, as this may facilitate the transfer of these ARGs [24, 53]. Lactic acid bacteria are generally regarded as nonpathogenic and beneficial microorganisms widely employed in food production and probiotic formulations [54]. Within *Lactococcus*, Lsa(F) is primarily plasmid-borne or has become stably integrated into specific chromosomal loci. Given these characteristics, the potential risk for horizontal transfer of the Lsa(F) gene to foodborne pathogens remains a matter of concern. Importantly, our results confirm that the Lsa(F) gene can be horizontally transferred from *S. parasuis* to *S. suis*, highlighting its potential for interspecies transmission.

The movement of many genes encoding the ABC-F family proteins between different MGEs is closely associated with IS elements, which typically appear in pairs flanking the resistance gene to form a compound transposon [14, 15], or individually positioned either upstream or downstream of the gene [55]. For instance, the optrA gene is frequently flanked by two copies of IS*1216E* elements oriented in the same direction; these two copies of IS*1216E* elements can form a translocation unit capable of moving between different MGEs [15, 40]. We found the presence of diverse IS elements from different families adjacent to the Lsa(F) gene; however, only a limited number of strains exhibited the gene flanked on both sides by homologous IS elements from the IS*3* or IS*6* families. Notably, the IS*Lll1* element, belonging to the IS982 family, is found in nearly all downstream regions of Lsa(F), suggesting it may promote the transposition of this gene. A pertinent example is the IS*1592* element, also from the IS*982* family, which is situated within a plasmid harboring the florfenicol and chloramphenicol resistance gene *floR*. This plasmid is believed to have arisen from several recombination events, in which IS*1592* may have played a role [56]. Consequently, it is hypothesized that the emergence of Lsa(F) within different MGEs may similarly be mediated by IS*Lll1*, and that Lsa(F) could be further disseminated to other bacteria via MGEs.

In summary, this study firstly identified the novel PLS_A_ resistance gene Lsa(F) in an animal-derived isolate of *S. parasuis*. The emergence of the novel IS*Lll1*-flanked Lsa(F) indicates its potential for mobility. The Lsa(F) gene is frequently associated with various MGEs. Despite the present detection rate remaining comparatively low, this genetic context could significantly increase the risk of its transmission. Importantly, Lsa(F) often coexists with other ARGs; thus, ongoing monitoring of the prevalence and dissemination of this gene is essential from a One Health perspective.

## Supplementary Information

**Additional file 1**. **The information of** ***Streptococcus parasuis*** **clinical strains in this study.**

**Additional file 2. Bacterial strains, plasmids, and primers used in this study.**

**Additional file 3**.** MICs for** ***S. parasuis*** **SFJ45,** ***S. suis*** **P1/7RF, and the transconjugants.**

**Additional file 4. Identity comparison of Lsa variant proteins**.

**Additional file 5.** **Information of the** **Lsa (F)-positive strains in GenBank.**

**Additional file 6. Genomic phylogenetic tree of Lsa(F)-positive** ***S***. ***suis* strains.** A WGS-based phylogenetic tree was constructed from 18 genomes, including *S. suis*, *S. parasuis*, and *Streptococcus agalactiae*. All 13 Lsa(F)-positive strains (red font indicates) originally deposited as *S. suis* clustered within the same clade as the *S. parasuis* reference strain SUT-286, whereas the *S. suis* reference strain BM407 formed a distinct lineage. This phylogenetic grouping suggests that these GenBank-deposited Lsa(F)-positive strains may have been misidentified and are *S. parasuis* rather than *S. suis.*

**Additional file 7. Comparative analysis of Lsa(F) and Lsa(F)-like.** The Lsa(F)-like protein consists of 522 amino acids and shares structural similarities with Lsa(F), exhibiting an identity of 81.2%.

**Additional file 8. The Lsa(F) gene located on short contigs in *Streptococcus* species.** Open reading frames (ORFs) are shown as arrows indicating the transcription direction. Homologous gene clusters in different strains are shaded in grey.

## Data Availability

All data supporting the findings of this study are available within the paper and its Supplementary Information.
