# Supplementary material for: Characterization of a novel gene, Lsa(F), conferring resistance to pleuromutilins, lincosamides and streptogramin A in Streptococcus parasuis
Source: Vet Res. 2026 Jul 7;57:122. doi: 10.1186/s13567-026-01784-0 (PMC13339394; doi:10.1186/s13567-026-01784-0)
Supplement: Supplementary file 1 — Additional file 1. The information of Streptococcus parasuis clinical strains in this study. [file 13567_2026_1784_MOESM1_ESM.pdf]

Additional file 1. The information of *Streptococcus parasuis* clinical strains used in this study.

Strains	Host	Year	Region	TIA MIC (mg/L)	Pleuromutilins-associated ARGs	*lsa*(F)-associated MGEs	WGS	Accession	Ref.
AH0906	Diseased pig	2009	Anhui	128	*lsa*(E)	–	Y	JANFLX01	[53]
BSJ48 *	Healthy pig	2016	Jiangsu	64	*lsa*(F)	GI_*fda*	Y	JANFMD01	[15]
HCJ16 *	Healthy pig	2016	Jiangsu	1	–	–	Y	JANFMG01	[15]
HCJ19 *	Healthy pig	2016	Jiangsu	64	*lsa*(E)	–	Y	JANFMH01	[15]
HCJ31 *	Healthy pig	2016	Jiangsu	64	*lsa*(F)	ICE_*fda*	Y	JAIMEP01	[15]
YTJ2 *	Healthy pig	2016	Jiangsu	≤0.5	–	–	Y	JAIMDU01	[15]
FHJ40	Healthy pig	2017	Jiangsu	≤0.5	–	–	N	–	This study
SFB2 *	Diseased pig	2017	Jiangsu	64	*lsa*(F)	ICE_*fda*	Y	JANFML01	[15]
SFJ35 *	Healthy pig	2017	Jiangsu	32	*lsa*(F), *cfr*	dICE_*fda*	Y	JANFMN01	[15]
SFJ44 *	Healthy pig	2017	Jiangsu	64	*lsa*(F), *cfr*	dICE_*fda*	Y	CP031970	[15]
SFJ45	Healthy pig	2017	Jiangsu	128	*lsa*(F)	CIME_*fda*	Y	CP102747	[22]
WZB2	Diseased pig	2017	Jiangsu	32	*lsa*(F)	ND	N	–	This study
FJMX9	Diseased pig	2018	Fujian	2	–	–	N	–	This study
FJNP8	Diseased pig	2018	Fujian	16	*lsa*(E)	–	N	–	This study
JS7	Diseased pig	2018	Zhejiang	16	*lsa*(E)	–	N	–	This study
JSWYW1	Diseased pig	2018	Zhejiang	128	*lsa*(F)	ND	N	–	This study
JXB6	Diseased pig	2018	Jiangxi	1	–	–	N	–	This study

2206xyZF3-1SS	Diseased pig	2022	Guizhou	≤0.5	–	–	N	–	This study
2206xyZF5SS	Diseased pig	2022	Guizhou	≤0.5	–	–	N	–	This study
JXHYB6SS1	Diseased pig	2022	Jiangxi	64	*lsa*(E)	–	N	–	This study
JXHYB6SS2	Diseased pig	2022	Jiangxi	64	*lsa*(E)	–	N	–	This study
SPS24010	Diseased pig	2024	Guizhou	64	*lsa*(F)	ND	N	–	This study
SPS1	Diseased pig	2025	Hunan	128	*lsa*(E)	–	N	–	This study
SPS2	Diseased pig	2025	Hunan	128	*lsa*(E)	–	N	–	This study
SPS921	Healthy pig	2025	Jiangsu	64	*lsa*(F)	ND	N	–	This study
WUSS033	Healthy pig	Unknown	Unknown	64	*lsa*(E)	–	N	–	This study

* Although initially misidentified as *Streptococcus suis* in GenBank, core genome phylogenetic analysis confirmed these strains as *S. parasuis* (see Additional file 6).

–, not applicable; ND, no detection.
