# Supplementary material for: Characterization of a novel gene, Lsa(F), conferring resistance to pleuromutilins, lincosamides and streptogramin A in Streptococcus parasuis
Source: Vet Res. 2026 Jul 7;57:122. doi: 10.1186/s13567-026-01784-0 (PMC13339394; doi:10.1186/s13567-026-01784-0)
Supplement: Supplementary file 2 — Additional file 2. Bacterial strains, plasmids, and primers used in this study. [file 13567_2026_1784_MOESM2_ESM.pdf]

Additional file 2. Bacterial strains, plasmids, and primers used in this study.

Strains	Characteristics		Ref.
Streptococcus suis			This Study
P1/7	*Streptococcus suis* standard strain		
P1/7RF	P1/7 with rifampicin and fusidic acid resistance		
P1/7::pSET2	pSET2-carrying strain P1/7		
P1/7::pSET2-*lsa*(F)	pSET2-*lsa*(F)-carrying strain P1/7		
Staphylococcus aureus			This Study
RN4220	Engineered clone strain with mutation in the *sau1 hsdR* gene		
RN4220::pSET2	pSET2-carrying strain RN422		
RN4220::pSET2-*lsa*(F)	pSET2-*lsa*(F)-carrying strain RN422		
Escherichia coli			This Study
DH5α	Engineered clone strain		
DH5α::pSET2	pSET2-carrying strain DH5α		
DH5α::pSET2-*lsa*(F)	pSET2-*lsa*(F)-carrying strain DH5α		
**Plasmids**	**Characteristics**		**Ref.**
pSET2	*Streptococcus-Escherichia coli* shuttle vector; Spc^R		[28]
pSET2-*lsa*(F)	*lsa*(F)-carrying vector pSET2		This Study
**Primers**	**Sequence (5'→3')**	**Product size (bp) / Tm (°C)**	**Ref.**
**Identification of *S. parasuis* (SPS) and *S. suis* (SS) species**			
SPS-recN	F: CAACTGCTGGATAGTTTCGG, R: GTCTGGCTGAGCTTAATTGG	679 / 54	[20]
SS-recN	F: CTACAAACAGCTCTCTTCT, R: ACAACAGCCAATTCATGGCGTGATT	336 / 55	[21]

**Detection of resistance genes *cfr*, *cfr*(D), *lsa*(E), and *lsa*(F)**			
cfr	F: TGAAGTATAAAGCAGGTTGGGAGTCA, R: ACCATATAATTGACCACAAGCAGC	746 / 48	[26]
cfr(D)	F: AGAAGTCGCAACAAGTGAGGA, F: GCAACTGCATGAGTCAAAGAA	595 / 55	[27]
lsa(E)	F: TGTCAAATGGTGAGCAAACG, R: TGTAAAACGGCTTCCTGATG	496 / 58	[25]
lsa(F)	F: AATTGCGGCTGTTGTTGGAC, R: TGCTGCCCTTGACTCATTGT	276 / 57	This Study
**Amplification of the complete ORF of *lsa*(F), for cloning onto pSET2**			
pSET2-lsa(F)	F: caggtcgactctagaggatccAATGAACGAGGTAAGGTCTATTGAAA,<br>R: aaaacgacggccagtgaattcGTCATGGCTAAATAATCAACTAGCAAT	2185 / 65	This Study
**Detection of resistance gene *spc* in pSET2 and its derived-plasmid**			
spc	F: CGGAAATTATGACTTAGAG, R: GGAGAAGATTCAGCCACT	211 / 53	This Study

Note: The PCR reaction system include, in volume 25 μL of 2 × Rapid Taq Master Mix (for routine amplification) or 2 × Phanta Max Master Mix (for high-fidelity amplification) (Vazyme, China), 1 μL of DNA template, and 2 μL each of forward and reverse primers (10 μM), and RNase-free water was added to achieve a total volume of 50 μL. The thermal cycling conditions were as follows: 3 min at 95°C, 32 cycles of 15 s at 95 °C, 10 s at Tm °C, extension time at 72 °C, and a final incubation of 5 min at 72°C. The extension time per cycle varied according to the polymerase used: for 2 × Rapid Taq Master Mix, the extension speed was 15 s/kb, whereas for 2 × Phanta Max Master Mix, the extension speed ranged from 30 to 60 s/kb.
