# Supplementary material for: Characterization of a novel gene, Lsa(F), conferring resistance to pleuromutilins, lincosamides and streptogramin A in Streptococcus parasuis
Source: Vet Res. 2026 Jul 7;57:122. doi: 10.1186/s13567-026-01784-0 (PMC13339394; doi:10.1186/s13567-026-01784-0)
Supplement: Supplementary file 3 — Additional file 3. MICs for S. parasuis SFJ45, S. suis P1/7RF, and the transconjugants. [file 13567_2026_1784_MOESM3_ESM.pdf]

**Additional file 3. MICs for *S. parasuis* SFJ45, *S. suis* P1/7RF, and the transconjugants.**

| **Strain** [a] | **Minimum inhibitory concentration (mg/L)** [b] | | | | | | | |
|---|---|---|---|---|---|---|---|---|
| | TIA | VAL | FFC | LZD | ERY | LIN | CLI | TET |
| SFJ45 | 128 | 128 | 64 | 4 | >128 | >128 | >128 | 128 |
| P1/7RF | 0.5 | ≤0.5 | 1 | 0.25 | 0.12 | ≤0.5 | ≤0.5 | 2 |
| TC-SFJ45-I | 1 | ≤0.5 | 32 | 4 | 2 | 128 | 64 | 2 |
| **TC-SFJ45-IC** | **64** | **32** | 32 | 2 | 2 | 64 | 32 | 4 |

[a] The drug selection markers were 4 mg/L lincomycin, 50 mg/L rifampicin, and 50 mg/L fusidic acid. Transfer frequency was calculated by CFUs of transconjugants/donors. The total transfer frequency was 1.01±2.71, and 53.3% of transconjugants were identified as TC-SFJ45-I, while the remaining 46.7% were classified as TC-SFJ45-IC, as determined by PCR analysis.

[b] TIA, Tiamulin; VAL, Valnemulin; FFC, florfenicol; LZD, linezolid; ERY, erythromycin; LIN, lincomycin; CLI, clindamycin; TET, tetracycline.
