# Supplementary material for: Characterization of a novel gene, Lsa(F), conferring resistance to pleuromutilins, lincosamides and streptogramin A in Streptococcus parasuis
Source: Vet Res. 2026 Jul 7;57:122. doi: 10.1186/s13567-026-01784-0 (PMC13339394; doi:10.1186/s13567-026-01784-0)
Supplement: Supplementary file 4 — Additional file 4. Identity comparison of Lsa variant proteins. [file 13567_2026_1784_MOESM4_ESM.pdf]

Additional file 4. Identity comparison of Lsa variant proteins.

Identity (%)	Lsa(A)	Lsa(B)	Lsa(C)	Lsa(D)	Lsa(E)	Lsa(F)
Lsa(A)	—	41.48	44.29	52.91	44.40	52.83
Lsa(B)	41.48	—	53.46	41.25	61.38	42.32
Lsa(C)	44.29	53.46	—	43.46	53.86	42.50
Lsa(D)	52.91	41.25	43.46	—	41.45	58.70
Lsa(E)	44.40	61.38	53.86	41.45	—	41.94
Lsa(F)	52.83	42.32	42.50	58.70	41.94	—

The GenBank accession numbers of the Lsa proteins are: Lsa(A) (AAO43110), Lsa(B) (NP_899166), Lsa(C) (AEA37904), Lsa(D) (AXF35727), Lsa(E) (AAL05553), and Lsa(F) (WFB92308).
