# Supplementary material for: Characterization of a novel gene, Lsa(F), conferring resistance to pleuromutilins, lincosamides and streptogramin A in Streptococcus parasuis
Source: Vet Res. 2026 Jul 7;57:122. doi: 10.1186/s13567-026-01784-0 (PMC13339394; doi:10.1186/s13567-026-01784-0)
Supplement: Supplementary file 5 — Additional file 5. Information of the Lsa (F)-positive strains in GenBank. [file 13567_2026_1784_MOESM5_ESM.pdf]

**Additional file 5. Information of the *lsa*(F)-positive strains in GenBank.**

| Strain [a] | Accession | Length (bp) | Year | Region | Host | Location [b] | Identity to Lsa(F) (%) [d] | MGE-encoded AMR genes |
|---|---|---|---|---|---|---|---|---|
| ***Streptococcus parasuis*** | | | | | | | | |
| FZ1 | CP170759 | 2054729 | 2021 | Shandong, China | Diseased pig | dICE_*fda* | 100.00 | *lsa*(F), *optrA*, *erm*(B), *aac(6')-aph(2'')*, *ant(6)-I*, *ant(9)-Ia* |
| FZ2 | CP170760 | 2032338 | 2021 | Shandong, China | Diseased pig | dICE_*fda* | 99.81 | *lsa*(F), *optrA*, *erm*(A), *erm*(B), *aac(6')- aph(2'')*, *ant(6)-I*, *ant(9)-Ia* |
| H35 | CP076721 | 2186318 | 2018 | China | Pig | CIME_*fda* | 99.62 | *lsa*(F), *mdt*(A) |
| SFJ45 | CP102747 (This study) | 2015398 | 2017 | Jiangsu, China | Pig | CIME_*fda* | 100.00 | *lsa*(F), *optrA*, *erm*(B), *mdt*(A) |
| SS17 | CP090522 | 1984594 | 2021 | Guangdong, China | Pig | CIME_*fda* | 100.00 | two copies of *lsa*(F) |
| SS20 | CP086728 | 1961908 | 2021 | Guangdong, China | Pig | CIME_*fda* | 100.00 | two copies of *lsa*(F) |
| ***Streptococcus suis* [a]** | | | | | | | | |
| 15338-02821 | DASEWR010000039 | 8122 | < 2023 | Viet Nam | Pig | unknown | 100.00 | *lsa*(F) |
| 15339-02824 | DASEXC010000003 | 146972 | < 2023 | Viet Nam | Pig | Fragment in downstream of *nemA* | 100.00 | *lsa*(F), *ant(6)-I*, *ant(9)-Ia* |
| 15367-09511 | DASEWV010000025 | 13109 | < 2023 | Viet Nam | Pig | unknown | 100.00 | *lsa*(F), *mdt*(A) |
| 15368-09512 | DASEWX010000009 | 81152 | < 2023 | Viet Nam | Pig | Fragment in upstream of *SUT286_1793* | 99.81 | *lsa*(F), *mdt*(A) |
| F-02-3-TS-05-SS-C4 | DBABDT010000032 | 16710 | 2016-2017 | Myanmar | Pig | unknown | 99.81 | *lsa*(F) |
| BS11F | JAAVJT010000036 | 3013 | 2017 | Sichuan, China | Pig | unknown | 100.00 | *lsa*(F) |
| SC1B19 | JABTZG010000023 | 7861 | 2017 | Sichuan, China | Pig | unknown | 99.62 | *lsa*(F) |
| SC2B29-1 | JABTZF010000023 | 7861 | 2017 | Sichuan, China | Pig | unknown | 99.62 | *lsa*(F) |
| BSJ48 | JANFMD010000030 (This study) | 8810 | 2016 | Jiangsu, China | Healthy pig | GI_*fda* | 99.81 | *lsa*(F), *optrA*, *erm*(A), *erm*(B), *aac(6')- aph(2'')*, *ant(6)-I*, *ant(9)-Ia* |

| | | | | | | | | |
|---|---|---|---|---|---|---|---|---|
| HCJ31 | JAIMEP010000047 (This study) | 8808 | 2016 | Jiangsu, China | Healthy pig | ICE_*fda* | 100.00 | *lsa*(F), *erm*(B), *ant(6)-I*, *ant(9)-Ia*, *mdt*(A) |
| SFB2 | JANFML010000018 (This study) | 42584 | 2017 | Jiangsu, China | Diseased pig | ICE_*fda* | 99.81 | *lsa*(F), *lnu*(D), *ant(6)-I*, *ant(9)-Ia*, *mdt*(A) |
| SFJ35 | JANFMN010000021 (This study) | 10911 | 2017 | Jiangsu, China | Healthy pig | dICE_*fda* | 100.00 | *lsa*(F), *optrA*, *ant(9)-Ia* |
| SFJ44 | CP031970 (This study) | 2026249 | 2017 | Jiangsu, China | Healthy pig | dICE_*fda* | 100.00 | *lsa*(F) |
| ***Streptococcus pluranimalium*** | | | | | | | | |
| pxr-18 | JBLVTX010000018 | 7612 | | Jiangsu, China | | Plasmid fragment | 99.62 | *lsa*(F) |
| SS-15 | CP161816 | 29824 | 2023 | Jilin, China | Pig | Plasmid | 99.62 | *lsa*(F), *lnu*(A) |
| ***Lactococcus lactis*** | | | | | | | | |
| 752 | RIGJ01000064 | 44372 | 2005 | Mali | Milk | Chr. | 98.73 | *lsa*(F) |
| 756 | RIGH01000012 | 292529 | 2005 | Mali | Milk | Chr. | 99.62 | *lsa*(F) |
| 1040 | RIGD01000027 | 185318 | 2007 | Somalia | Camel milk | Chr. | 98.86 | *lsa*(F) |
| OSY-92 | JBDPLO010000079 | 3516 | 2022 | USA | Cheese | Chr. | 99.62 | *lsa*(F) |
| P30-3 | JARQCH010000018 | 25035 | 2022 | China | Pig | Plasmid fragment | 99.43 | *lsa*(F) |
| P83-3 | JARQCG010000027 | 9546 | 2022 | China | Pig | Plasmid fragment | 99.62 | *lsa*(F) |
| PHM5-37 2 | JALBCN010000002 | 478886 | 2014 | India | Zea mays | Chr. | 99.62 | *lsa*(F) |
| SS45 | JABCRL010000032 | 6838 | 2019 | China | Pig | unknown | 99.62 | *lsa*(F) |
| SZ1-15 | JBEFPR010000031 | 6877 | 2023 | China | Hospital sewage | unknown | 99.61 | *lsa*(F), *vat*(E) |
| Y38-1 | JARQDK010000027 | 10038 | 2022 | China | Fish | unknown | 99.62 | *lsa*(F) |
| Y67-2 | JARQCQ010000016 | 23158 | 2022 | China | Fish | Plasmid fragment | 99.62 | *lsa*(F) |
| ***Lactococcus lactis* subsp. *lactis*** | | | | | | | | |
| Dephy 1 | CBUJ010000038 | 5059 | < 2013 | | | unknown | 99.62 | *lsa*(F) |

| | | | | | | | | |
|---|---|---|---|---|---|---|---|---|
| EIP33 | CP086073 | 66370 | 2017 | France | Goat | Plasmid | 99.62 | *lsa*(F) |
| 14B4B [c] | CP028160 | 2579381 | 2014 | USA | Almond drupe | Chr. | 81.21 | *lsa*(F)-like |
| ***Lactococcus sp.*** | | | | | | | | |
| NH2-7C | CP124538 | 2595399 | 2021 | Thailand | Meat | Chr. | 99.62 | *lsa*(F) |
| UCCL620 | PP556496 | 42038 | < 2024 | | Milk | Plasmid | 99.62 | *lsa*(F) |
| ***Lactococcus cremoris*** | | | | | | | | |
| AM1 [c] | WJVE01000041 | 73241 | 2015 | | | Chr. | 80.68 | *lsa*(F)-like |

[a] The comparative genomics analysis indicates that the *lsa*(F)-positive strain of *Streptococcus suis* is, in fact, *Streptococcus parasuis* (Details are provided in Additional file 6).

[b] The plasmid fragment suggests that a Rep3 or RepB family plasmid replication protein is located either upstream or downstream of *lsa*(F), leading to the inference that *lsa*(F) is situated on the plasmid. Furthermore, the chromosomal genes *nemA* and *SUT286_1793* were identified in proximity to *lsa*(F) in two strains.

[c] The rows marked with a grey background indicate the absence of the *lsa*(F) gene in strains 14B4B and AM1, which instead harbor the *lsa*(F)-like gene. In fact, many *Lactococcus* strains carry the *lsa*(F)-like gene, with these two strains serving as representative examples in *L. lactis* and *L. cremoris.*

[d] The Lsa(F) sequence refers to the Lsa(F) sequence found in *S. parasuis* strain SFJ45.
