# Supplementary figures and images for: Characterization of a novel gene, Lsa(F), conferring resistance to pleuromutilins, lincosamides and streptogramin A in Streptococcus parasuis
Source: Vet Res. 2026 Jul 7;57:122. doi: 10.1186/s13567-026-01784-0 (PMC13339394; doi:10.1186/s13567-026-01784-0)

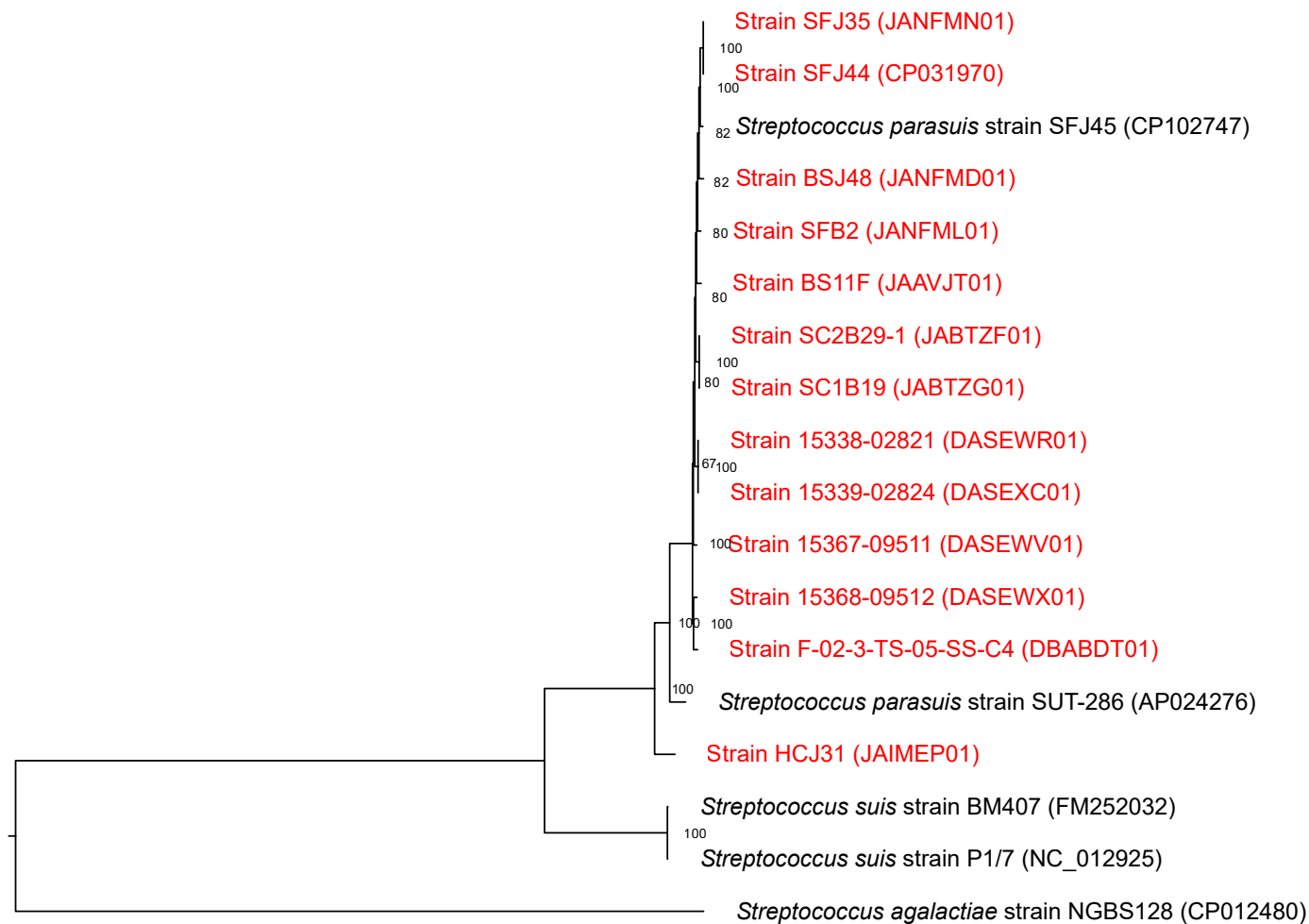

0.08

Supplement: Supplementary file 6 — Additional file 6. Genomic phylogenetic tree of Lsa(F)-positive S. suis strains. A WGS-based phylogenetic tree was constructed from 18 genomes, including S. suis, S. parasuis, and Streptococcus agalactiae. All 13 Lsa(F)-positive strains (red font indicates) originally deposited as S. suis clustered within the same clade as the S. parasuis reference strain SUT-286, whereas the S. suis reference strain BM407 formed a distinct lineage. This phylogenetic grouping suggests that these GenBank-deposited Lsa(F)-positive strains may have been misidentified and are S. parasuis rather than S. suis. [file 13567_2026_1784_MOESM6_ESM.pdf]

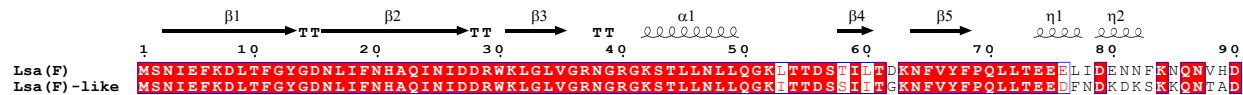

Walker A1

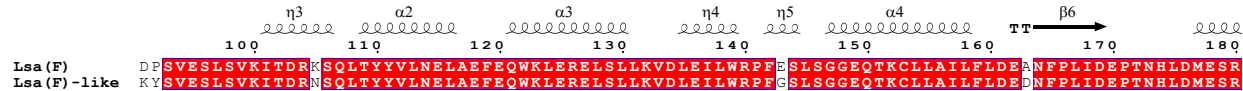

ABC Signature 1

Walker B1

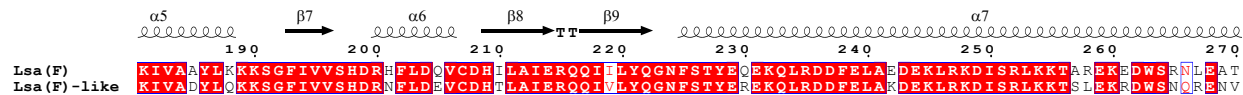

H-loop 1

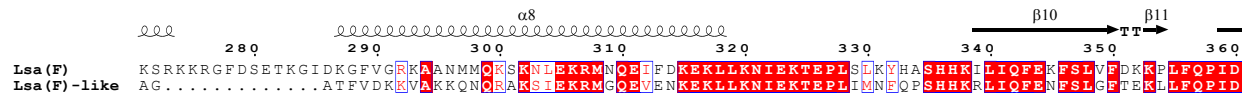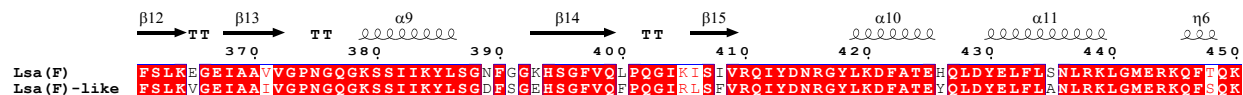

Walker A2

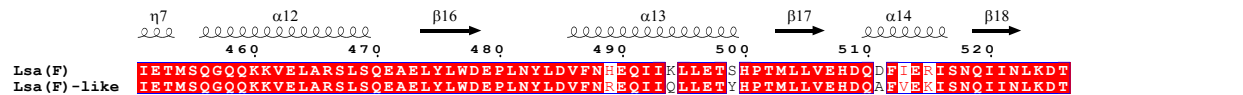

ABC Signature 2

Walker B2

H-loop 2

Supplement: Supplementary file 7 — Additional file 7. Comparative analysis of Lsa(F) and Lsa(F)-like. The Lsa(F)-like protein consists of 522 amino acids and shares structural similarities with Lsa(F), exhibiting an identity of 81.2%. [file 13567_2026_1784_MOESM7_ESM.pdf]
