# Supplementary material for: Characterization of a novel gene, Lsa(F), conferring resistance to pleuromutilins, lincosamides and streptogramin A in Streptococcus parasuis
Source: Vet Res. 2026 Jul 7;57:122. doi: 10.1186/s13567-026-01784-0 (PMC13339394; doi:10.1186/s13567-026-01784-0)
Supplement: Supplementary file 8 — Additional file 8. The Lsa(F) gene located on short contigs in Streptococcus species. Open reading frames (ORFs) are shown as arrows indicating the transcription direction. Homologous gene clusters in different strains are shaded in grey. [file 13567_2026_1784_MOESM8_ESM.pdf]

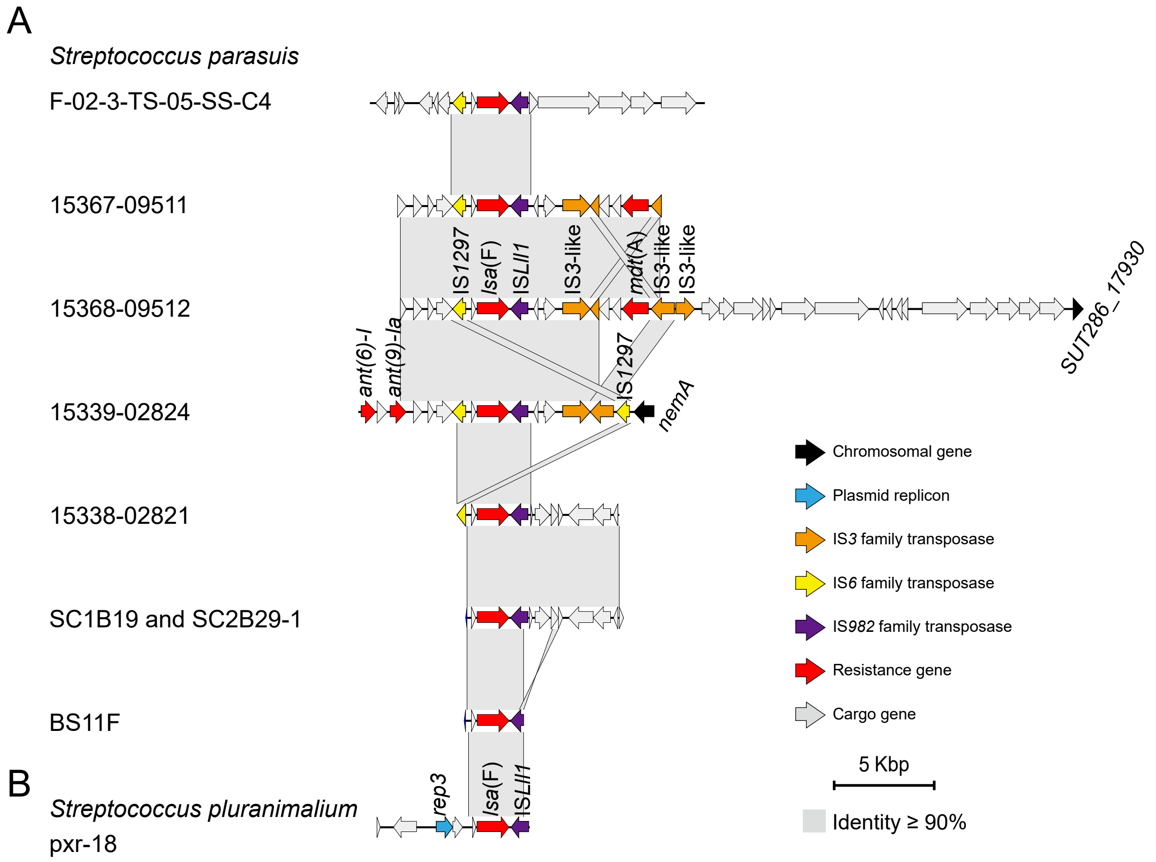

A
Streptococcus parasuis
F-02-3-TS-05-SS-C4
15367-09511
15368-09512
15339-02824
15338-02821
SC1B19 and SC2B29-1
BS11F
IS1297
lsa(F)
ISLII1
IS3-like
mdt(A)
IS3-like
IS3-like
SUT286_17930
ant(6)-I
ant(9)-Ia
IS1297
nemA
B
Streptococcus pluranimalium
pxr-18
rep3
lsa(F)
ISLII1
Chromosomal gene
Plasmid replicon
IS3 family transposase
IS6 family transposase
IS982 family transposase
Resistance gene
Cargo gene
5 Kbp
Identity ≥ 90%
